# Agravity up to infinite energy

**DOI:** 10.1140/epjc/s10052-018-5588-4

**Published:** 2018-02-10

**Authors:** Alberto Salvio, Alessandro Strumia

**Affiliations:** 1 Theoretical Physics Department, CERN, Geneva, Switzerland; 2 Dipartimento di Fisica dell’Universitá di Pisa and INFN, Pisa, Italy

## Abstract

The self-interactions of the conformal mode of the graviton are controlled, in dimensionless gravity theories (agravity), by a coupling $f_0$ that is not asymptotically free. We show that, nevertheless, agravity can be a complete theory valid up to infinite energy. When $f_0$ grows to large values, the conformal mode of the graviton decouples from the rest of the theory and does not hit any Landau pole provided that scalars are asymptotically conformally coupled and all other couplings approach fixed points. Then agravity can flow to conformal gravity at infinite energy. We identify scenarios where the Higgs mass does not receive unnaturally large physical corrections. We also show a useful equivalence between agravity and conformal gravity plus two extra conformally coupled scalars, and we give a simpler form for the renormalization group equations of dimensionless couplings as well as of massive parameters in the presence of the most general matter sector.

## Introduction

The idea that scalars, like the Higgs, must be accompanied by new physics that protects their lightness from power-divergent quantum corrections led to the following view of mass scales in nature: the weak scale is the supersymmetric scale, and the Planck scale is the string scale. The non-observation of supersymmetric particles around the weak scale challenged this scenario, leading to the alternative idea that only physical corrections to scalar masses must satisfy naturalness. Namely, extra new particles with mass $M_{\mathrm{extra}}$ and coupling $g_{\mathrm{extra}}$ to the Higgs, must satisfy

1 $$\begin{aligned} \delta M_h \sim g_{\mathrm{extra}} M_{\mathrm{extra}} \lesssim M_h. \end{aligned}$$

A rationale for ignoring power-divergent corrections is the following. The one-loop quantum correction to the masses of scalars, vectors and of the graviton is power divergent, showing the dangers of attributing physical meaning to power-divergent corrections. A cut-off (such as string theory) that knows that vector and graviton masses are protected by gauge invariance can keep them to zero, while giving a large correction to scalar masses. A less smart cut-off (such as dimensional regularization) can be blind to the difference, and set to zero all power divergences. The simplest cut-off with this property is no cut-off: a theory where all renormalizable couplings flow up to infinite energy without hitting Landau poles.

The above arguments motivate the following scenario: if nature is described at fundamental level by a dimensionless Lagrangian, all power-divergent quantum corrections—being dimensionful—must be interpreted as vanishing. Taking gravity into account, the most general dimensionless action in $3+1$ space-time dimensions contains gauge couplings, Yukawa couplings, scalar quartics, non-minimal $\xi$-couplings between scalars and gravity and, in the purely gravitational sector, two dimensionless gravitational couplings, $f_0$ and $f_2$, analogous to gauge couplings:

2 $$\begin{aligned} S=\int \mathrm{{d}}^4x\,\sqrt{|\det g|} \bigg [ \frac{R^2}{6f_0^2} + \frac{\frac{1}{3} R^2 - R_{\mu \nu }^2}{f_2^2} + \mathscr {L}_\mathrm{matter}\bigg ], \end{aligned}$$

where $\mathscr {L}_{\mathrm{matter}}$ corresponds to the part of the Lagrangian that depends on the matter fields, with dimensionless parameters only. This theory [1] is renormalizable, as suggested in [2] and formally proven in [3]. The weak scale, the QCD scale and the Planck scale can be dynamically generated [4] from vacuum expectation values or from condensates. Perturbative dimensionless theories automatically give slow-roll inflation [4–9] (see also refs. [10, 11] for related studies).

However, Eq. (2) means that four derivatives act on the graviton: thereby some graviton components have a negative kinetic term.^1^ Classically the theory in (2) is sick [13]: the energy is unbounded from below. A sensible quantum theory might exist, analogously to what happens with fermions: their classical energy is negative, but their quantum theory is sensible.^2^ We will not address this problem here.

We will here study whether this theory can flow up to infinite energy. The Quantum Field Theory (QFT) part can have this property. Realistic TeV-scale extensions of the Standard Model (SM) can be asymptotically free [23, 24], and it is not known whether the SM itself can be asymptotically safe, in a non-perturbative regime [25]. The gravitational coupling $f_2$ is asymptotically free. The difficulty resides in the coupling $f_0$: a small $f_0$ grows with energy, until it becomes large.

In this paper we will show that, despite this, the theory can flow up to infinite energy, in an unusual way. In Sect. 2 we present an alternative formulation of agravity that makes it easier to compute its renormalization group equations (RGE): $f_0$ becomes the quartic of a special scalar, the conformal mode of the agraviton. Then a large $f_0$ means that the conformal mode of the agraviton gets strongly self-coupled. The rest of the theory decouples from it, if at the same time all scalars become conformally coupled, namely if all $\xi$ parameters run to $-1/6$, and all the other couplings reach ultraviolet (UV) fixed points, where all $\beta$-functions vanish.

In Sect. 4 we isolate the conformal mode of the graviton and show that its strong dynamics is such that $f_0$ does not hit a Landau pole. This means that the infinite-energy limit of agravity can be conformal gravity. The unusual phenomenon that allows one to reach infinite energy is that the conformal mode of the graviton fluctuates freely, but the rest of theory is not coupled to it: it becomes a gauge redundancy of a new local symmetry, Weyl symmetry. Since this symmetry is anomalous, conformal gravity cannot be the complete theory: going to lower energy the conformal model of the graviton starts coupling to the rest of the theory, which becomes agravity. This issue is discussed in Sect. 3. In Sect. 5 we propose scenarios where the Higgs mass does not receive unnaturally large corrections. Conclusions are given in Sect. 6. Finally, in the appendix we provide a new and simple expression for the one-loop RGE of all dimensionless parameters (Appendix A) as well as of all dimensionful parameters (Appendix B) in the presence of the most general matter sector, which was not studied before.

## Agravity

Allowing for generic scalars $\phi _a$ with generic dimensionless coupling $\xi _{ab}$ to gravity, $-\frac{1}{2} \xi _{ab}\phi _a\phi _b R$, the one-loop RGE for $f_0$ is [4, 26–28]

3 $$\begin{aligned} (4\pi )^2\frac{\mathrm{{d}} f_0^2 }{\mathrm{{d}}\ln \bar{\mu }}= & {} \frac{5}{3} f_2^4 + 5 f_2^2 f_0^2 + \frac{5}{6} f_0^4 \nonumber \\&+\frac{f_0^4}{12} (\delta _{ab}+6\xi _{ab})(\delta _{ab}+6\xi _{ab}) >0 \nonumber \\&\quad \text { for } f_0 \ll 1, \end{aligned}$$

where $\bar{\mu }$ is the renormalization scale in the modified minimal subtraction scheme (see also [29, 30] for a previous attempt to determine this RGE). This shows that, in all theories, $f_0$ is asymptotically free only for $f_0^2<0$. However, negative $f_0^2$ corresponds to a run-away potential [4, 6, 7], and this instability cannot be made harmless (or even beneficial for explaining dark energy) by invoking a small enough negative $f_0^2$, since tests of gravity exclude extra graviton components below 0.05 eV (see [31, 32] for attempts to have $f_0^2 < 0$). The fact that $f_0^2<0$ is phenomenologically problematic was already noted in [4], where it was pointed out that it leads to a tachyonic instability. Barring stabilization through background effects in cosmology, one needs $f_0^2>0$. But the one-loop RGE show that a small $f^2_0>0$ grows until it becomes non-perturbative.^3^

**Table 1:** Transformations of coordinates and fields under a Weyl transformation

		Dilatation	$\otimes$	Diffeomorphism	=	Weyl transformation
Coordinates	$\mathrm{{d}}x^\mu$	$e^\sigma \mathrm{{d}}x^\mu$		$e^{-\sigma } \mathrm{{d}}x^\mu$		$\mathrm{{d}}x^\mu$
Graviton	$g_{\mu \nu }$	$g_{\mu \nu }$		$e^{2\sigma } g_{\mu \nu }$		$e^{2\sigma } g_{\mu \nu }$
Scalars	$\phi$	$e^{-\sigma } \phi$		$\phi$		$e^{-\sigma } \phi$
Vectors	$V_{\mu }$	$e^{-\sigma } V_{\mu }$		$e^{\sigma } V_{\mu }$		$V_{\mu }$
Fermions	$\psi$	$e^{-3\sigma /2} \psi$		$\psi$		$e^{-3\sigma /2} \psi$

These RGE show peculiar features. Only scalars (not vectors nor fermions) generate $f_0$ at one-loop, and only if their $\xi$-couplings have a non-conformal value, $\xi _{ab}\ne -\,\delta _{ab}/6$. The $\xi$-couplings often appear in the RGE in the combination $\xi _{ab}+\delta _{ab}/6$, but not always. The coupling $f_0$ appears at the denominator in the RGE for the $\xi$-couplings [4].

The above features can be understood noticing that a new symmetry appears in the limit $f_0 \rightarrow \infty$ and $\xi _{ab} \rightarrow -\,\delta _{ab}/6$: the Weyl (or local conformal) symmetry. The Weyl symmetry is a local dilatation $\mathrm{{d}}x^\mu \rightarrow e^{\sigma (x)} \mathrm{{d}}x^\mu$ compensated by the special diffeomorphism $\mathrm{{d}}x^\mu \rightarrow e^{-\sigma (x)} \mathrm{{d}}x^\mu$ such that the coordinates $\mathrm{{d}}x^\mu$ remain unaffected. The various fields rescale under a dilatation as determined by their mass dimension, and they transform under a diffeomorphism as dictated by their Lorentz indices, as summarized in Table 1.

Agravity is invariant under global Weyl transformations: being dimensionless, it is invariant under global dilatations (for which $\sigma$ does not depend on *x*); being covariant, it is invariant under local diffeomorphisms.

Agravity is not invariant under local Weyl transformations. A generic dimensionless theory can be written in terms of the metric $g_{\mu \nu }$, real scalars $\phi _a$, Weyl fermions $\psi _j$ and vectors $V_A$ (with field strength $F_{\mu \nu }^A$). The action $S = \int \mathrm{{d}}^4x \sqrt{|\det g|}\, \mathscr {L}$ can be split as  where the first part is invariant under Weyl transformations,4and the second part,5is not invariant.^4^ To see this we will now perform a Weyl transformation,

6 $$\begin{aligned}&g_{\mu \nu }(x) \rightarrow e^{2\sigma (x)} g_{\mu \nu }(x),\quad \phi (x)\rightarrow e^{-\sigma (x)}\phi (x),\nonumber \\&\psi (x)\rightarrow e^{-3\sigma (x)/2}\psi (x), \qquad V_\mu \rightarrow V_\mu . \end{aligned}$$

This will also lead to an equivalent formulation of the theory.

### Equivalent formulations of agravity

The extra scalar field $\sigma (x)$, defined in (6), will be called the ‘conformal mode of the agraviton’; for the moment it is introduced as an extra gauge redundancy. We will comment on the corresponding gauge symmetry later on.

All terms in Eq. (4) are invariant under Weyl transformations. Since vectors and fermions appear only in Eq. (4), $\sigma$ does not couple to them. Only the terms that break Weyl symmetry give rise to interactions of $\sigma$. The transformation (6) leads to

7 $$\begin{aligned}&\sqrt{|\det g|} \rightarrow e^{4\sigma }\sqrt{|\det g|},\nonumber \\&\quad R \rightarrow e^{-2\sigma } (R - 6 e^{-\sigma }\Box e^\sigma ). \end{aligned}$$

Therefore, the Weyl-breaking part of the Lagrangian becomes8which is one simple way to rewrite , which will be used later on.

Another simple and useful form of  can be obtained from (8) as follows. We define $\Omega _L =e^\sigma$ and complete the square rewriting Eq. (8) as9Next we write the square as $A^2/6f_0^2 = -\frac{1}{6} f_0^2 \Omega _L^2\Omega _R^2 +\frac{1}{3} \Omega _R \Omega _L A$ by introducing an auxiliary field $\Omega _R$ with quadratic action, such that integrating it out gives back the original action. The resulting expression only contains the combination $\Omega _L\Omega _R$, that is, invariant under $\Omega _L\rightarrow t\Omega _L$, $\Omega _R\rightarrow \Omega _R/t$, which forms a SO(1,1) scale symmetry. Indeed, one can verify that SO(1,1) is broken by adding Lagrangian terms with dimensionful coefficients, such as the Einstein–Hilbert term or the cosmological constant, as done later in Eq. (40). Now, we can rewrite $\Omega _L \Omega _R$ in vectorial notation as $\Omega _L\Omega _R = \frac{1}{4} (\Omega _+^2 - \Omega _-^2) =\frac{1}{4} \vec \Omega ^2$ by going from the “light-cone basis” $\Omega _{L,R}$ to the $\Omega _\pm$ basis as $\Omega _L = t( \Omega _+ - \Omega _-)/{2}$ and $\Omega _R=(\Omega _+ + \Omega _-)/{2t}$ and defining the SO(1,1) vector $\vec \Omega = (\Omega _+, \Omega _-)$. Then the Weyl-breaking part of the action can be written in the final form10The non-trivial result is that *the Weyl-breaking part of the action has been rewritten as an extra Weyl-invariant action* involving the extra scalar SO(1,1) doublet $\vec \Omega$, which describes the conformal mode of the agraviton.

We have not (yet) imposed any constraint on the metric $g_{\mu \nu }$ after the transformation in Eq. (6); therefore we have a Weyl-type gauge invariance acting as

11 $$\begin{aligned}&g_{\mu \nu }(x) \rightarrow e^{-2\chi (x)}g_{\mu \nu }(x), \quad \phi (x)\rightarrow e^{\chi (x)}\phi (x),\nonumber \\&\psi (x)\rightarrow e^{3\chi (x)/2}\psi (x), \qquad V_\mu \rightarrow V_\mu \end{aligned}$$

where $\chi (x)$ is an arbitrary real function of *x*. The transformation $\sigma \rightarrow \sigma +\chi$ is equivalent to including $\Omega _L =e^\sigma$ and $\Omega _R$ among the scalars $\phi$. Therefore, agravity is equivalent to conformal gravity plus two extra conformally coupled scalars, $\Omega _+$ and $\Omega _-$.^5^ In the new formulation of agravity with the field $\vec \Omega$, the gravitational couplings $f_0$ and $\xi _{ab}$ have become scalar quartic couplings.

The formulations presented in this section certainly are equivalent at the classical level. At quantum level, the equivalence needs to take into account the anomalous transformation law of the path-integral measure, which amounts to adding an effective $\sigma$-dependent term in the action. This amounts to $\sigma$ starting to couple to terms that break scale invariance proportionally to their quantum $\beta$-functions. These extra couplings only affect RGE at higher loop orders, as we will discuss in Sect. 3.

It is now clear why the one-loop RGE for $f_0$, Eq. (3), does not receive contributions from fermions and vectors: $f_0^2$ is the quartic coupling of a neutral scalar with no Yukawa interactions. A positive $f_0^2$ corresponds to a positive quartic. Furthermore the symmetry SO(1,1) can be complexified into SO(2) by redefining $\Omega _- \rightarrow i \Omega _-$ without affecting the RGE at perturbative level: only non-perturbative large field fluctuations are sensitive to the difference. By defining an extended set of quartic couplings, $\lambda _\mathrm{ABCD}$, where the capital indices run such that the quartics that involve the two extra scalars $\vec \Omega$ are included, the generic RGE for the scalar quartics only, known in a generic QFT up to two loops, are 6

12 $$\begin{aligned}&\frac{\mathrm{{d}} \lambda _{ABCD}}{\mathrm{{d}}\ln {\bar{\mu }}} = \frac{1}{(4\pi )^2} \sum _\mathrm{perms}\frac{1}{8} \lambda _\mathrm{ABEF}\lambda _{EFCD}\nonumber \\&\quad + \frac{1}{(4\pi )^4}\bigg [\frac{\gamma }{2} \lambda _{ABCD}- \frac{1}{4}\sum _{\mathrm{perms}} \lambda _{ABEF}\lambda _{CEGH}\lambda _{DFGH} \bigg ]+\cdots ,\nonumber \\ \end{aligned}$$

where $\gamma = \Lambda _{AA}+\Lambda _{BB}+\Lambda _{CC} + \Lambda _{DD}$ (with $\Lambda _{AB} = \frac{1}{6} \lambda _{ACDE}\lambda _{BCDE}$) is the scalar wave-function renormalization, the sums run over the 4! permutations of *ABCD* and $\cdots$ is the contribution of the other couplings.

From Eq. (12) one can re-derive the one-loop RGE for $f_0$ and $\xi _{ab}$, computed as gravitational couplings in [4]. The two results agree. Furthermore, the same RGE acquire a simpler form if rewritten in terms of the $\lambda _{ABCD}$ coefficients. The RGE are explicitly written in Eq. (50) in Appendix A, and neither $f_0$ nor any other coupling appear anymore at the denominator in the RGE.

### The graviton propagator

A gravitational computation is now only needed to compute the part of the RGE involving $f_2$. So far the field $\sigma$, or $\vec \Omega$, has been introduced as an extra gauge redundancy. One can fix it by setting $\sigma =0$, going back to the original formulation where the full RGE were computed in [4]. In the rest of this section (which contains technical details used only for a double check of the main results) we show how one can choose an alternative convenient condition: that the fluctuation $h'_{\mu \nu }$ around the flat space of $g_{\mu \nu }$ *after* the transformation in Eq. (6) has vanishing trace, that is,

13 $$\begin{aligned} h' \equiv \eta ^{\mu \nu }h'_{\mu \nu } = 0. \end{aligned}$$

We have introduced a prime in $h'_{\mu \nu }$ to distinguish it from the fluctuation $h_{\mu \nu }$ around the flat space of the metric *before* transformation (6). The new variables $h'_{\mu \nu }$ and $\sigma$ are given in terms of the old ones (the trace $h\equiv \eta ^{\mu \nu } h_{\mu \nu }$ and the traceless part $h^{\mathrm{TL}}_{\mu \nu }\equiv h_{\mu \nu }-\eta _{\mu \nu }h/4$) by

14 $$\begin{aligned} e^{2\sigma } = 1+\frac{h}{4}, \qquad h'_{\mu \nu } = e^{-2\sigma }h^\mathrm{TL}_{\mu \nu }. \end{aligned}$$

The path-integral measure $Dg_{\mu \nu }\equiv Dh \, Dh^\mathrm{TL}_{\mu \nu }$ splits as $Dg_{\mu \nu } = Dh'_{\mu \nu }\,D\sigma = Dh'_{\mu \nu } D\vec \Omega$. We neglect here the Weyl anomaly because, as explained above, it does not affect the one-loop RGE.

In order to compute quantum effects, we consider the following convenient gauge fixing for the diffeomorphisms $x^\mu \rightarrow x^\mu +\xi ^\mu (x)$:

15 $$\begin{aligned} \partial ^{\mu }h'_{\mu \nu }=0, \end{aligned}$$

where we use the flat metric $\eta _{\mu \nu }$ to raise and lower the indices. This choice avoids kinetic mixing between $\sigma$ and $h'_{\mu \nu }$ and leads to a simple propagator of $h'_{\mu \nu }$

16 $$\begin{aligned} D'_{\mu \nu \,\rho \sigma } = -2f_2^2 \frac{ i}{k^4} P^{(2)}_{\mu \nu \rho \sigma }, \end{aligned}$$

where

17 $$\begin{aligned}&P^{(2)}_{\mu \nu \rho \sigma } = \frac{1}{2} T_{\mu \rho }T_{\nu \sigma } + \frac{1}{2} T_{\mu \sigma } T_{\nu \rho }-\frac{1}{3} T_{\mu \nu }T_{\rho \sigma },\nonumber \\&T_{\mu \nu } = \eta _{\mu \nu } - k_\mu k_\nu /k^2. \end{aligned}$$

To determine the Lagrangian of the Fadeev–Popov ghosts we have to perform the variation of $\partial ^{\mu }h'_{\mu \nu }$ with respect to diffeomorphisms, whose effect on $h_{\mu \nu }$ at the linear level in $\xi ^\mu$ is

18 $$\begin{aligned}&h_{\mu \nu } \rightarrow h_{\mu \nu } - (\partial _\mu \xi _\nu + \partial _\nu \xi _\mu ) \nonumber \\&- (h_{\alpha \mu }\partial _\nu +h_{\alpha \nu }\partial _\mu + (\partial _\alpha h_{\mu \nu }))\xi ^\alpha . \end{aligned}$$

The effect of diffeomorphisms on $h'_{\mu \nu }$ and $\sigma$ can be computed by first splitting Eq. (18) in its traceless and trace parts,

19 $$\begin{aligned}&h \rightarrow h -2 \partial _\mu \xi ^\mu - 2 h^\mathrm{TL}_{\alpha \mu }\partial ^\mu \xi ^\alpha -\frac{1}{2} h \partial _\mu \xi ^\mu - \xi ^\alpha \partial _\alpha h, \end{aligned}$$

20 $$\begin{aligned}&h^{\mathrm{TL}}_{\mu \nu } \rightarrow h^{\mathrm{TL}}_{\mu \nu } -\partial _\nu \xi _\mu -\partial _\mu \xi _\nu +\frac{1}{2}\eta _{\mu \nu } \partial _\alpha \xi ^\alpha \nonumber \\&\quad -\, h^{\mathrm{TL}}_{\alpha \mu } \partial _\nu \xi ^\alpha - h^{\mathrm{TL}}_{\alpha \nu } \partial _\mu \xi ^\alpha - \partial _\alpha h^{\mathrm{TL}}_{\mu \nu } \xi ^\alpha \nonumber \\&\quad +\,\frac{1}{2} \eta _{\mu \nu } h^{\mathrm{TL}}_{\alpha \beta } \partial ^\beta \xi ^\alpha -\frac{1}{4} h (\partial _\nu \xi _\mu +\partial _\mu \xi _\nu )\nonumber \\&\quad +\,\frac{1}{8} \eta _{\mu \nu } h \partial _\alpha \xi ^\alpha , \end{aligned}$$

and next by using Eq. (14) to express $h^\mathrm{TL}_{\mu \nu }$ and *h* in terms of $h'_{\mu \nu }$ and $\sigma$. The result is

21 $$\begin{aligned} e^{2\sigma }\rightarrow & {} e^{2\sigma } \left( 1- \frac{1}{2} \partial _{\mu }\xi ^\mu - \frac{1}{2} h'_{\mu \alpha }\partial ^{\mu }\xi ^\alpha -2\xi _\alpha \partial ^\alpha \sigma \right) , \end{aligned}$$

22 $$\begin{aligned} h'_{\mu \nu }\rightarrow & {} h'_{\mu \nu } -\partial _\nu \xi _\mu -\partial _\mu \xi _\nu +\frac{1}{2}\eta _{\mu \nu } \partial _\alpha \xi ^\alpha \nonumber \\&- h'_{\alpha \mu } \partial _\nu \xi ^\alpha - h'_{\alpha \nu } \partial _\mu \xi ^\alpha - \partial _\alpha h'_{\mu \nu } \xi ^\alpha +\frac{1}{2} h'_{\mu \nu } \partial _\alpha \xi ^\alpha \nonumber \\&+\frac{1}{2} \eta _{\mu \nu } h'_{\alpha \beta } \partial ^\beta \xi ^\alpha +\frac{1}{2} h'_{\mu \nu }h'_{\alpha \beta } \partial ^\beta \xi ^\alpha . \end{aligned}$$

Notice that the transformation law of $h'_{\mu \nu }$ is independent of $\sigma$: having used the gauge in Eq. (15) the Fadeev–Popov procedure does not generate any new coupling of $\sigma$ to the Fadeev–Popov ghosts.^7^ In conclusion, we have shown how to implement the gauge where the graviton is traceless.

## Conformal gravity

We return to our physical issue: the coupling $f_0$ is not asymptotically free. In Sect. 4 we will argue that $f_0$ grows with energy, becoming non-perturbative at $f_0 \sim 4\pi$ and continuing to grow up to $f_0\rightarrow \infty$ in the limit of infinite energy, such that the $R^2/6f_0^2$ term disappears from the action. In this section we show that this limit is well defined. It is precisely defined as agravity with parameters chosen such that all Weyl-breaking terms  in Eq. (5) vanish:

23 $$\begin{aligned} f_0 =\infty ,\qquad \xi _{ab} =- \frac{\delta _{ab}}{6} . \end{aligned}$$

The $R^2/6 f_0^2$ term provides the kinetic term for $\sigma$, the conformal mode of the agraviton. Thereby $\sigma$ fluctuates wildly in the limit $f_0 \rightarrow \infty$. Indeed, the agraviton propagator of [4] has a contribution proportional to $f_0^2$, which diverges as $f_0\rightarrow \infty$. Faddeev and Popov have shown how to deal with these situations: add an extra gauge fixing for the extra gauge redundancy appearing in conformal gravity, local Weyl transformations.

In general, conformal gravity is not a consistent quantum theory, because its Weyl gauge symmetry is anomalous. In a simpler language, the dimensionless couplings run with energy as described by their RGE.^8^ The theory is no longer scale invariant, and the conformal mode of the graviton couples to all non-vanishing $\beta$-functions. The Weyl-breaking terms of the agravity Lagrangian are generated back by quantum corrections. The consistent quantum theory is agravity. For this reason our work differs from articles where conformal gravity is proposed as a complete theory of gravity [39, 40].

Nevertheless, conformal gravity can be the consistent infinite-energy limit of agravity provided that all $\beta$-functions vanish at infinite energy: the theory must be asymptotically free or asymptotically safe, in other words all couplings other than $f_0$ have to reach a UV fixed point where all $\beta$-functions vanish, as we will see.

In this section we clarify these issues by computing the one-loop RGE of conformal gravity coupled to a generic matter sector, as in Eq. (4). The RGE can be obtained without performing any extra computation by using the perturbative equality obtained in the previous section: agravity is equivalent to conformal gravity plus two extra scalars, $\vec \Omega$. In the other direction, this means that conformal gravity has the same RGE as agravity *minus* two scalars. Thereby the RGE for $f_2$ in conformal gravity is obtained by substituting $N_s\rightarrow N_s-2$ in (50a) obtaining

24 $$\begin{aligned} (4\pi )^2\frac{\mathrm{{d}}f_2^2}{\mathrm{{d}}\ln \bar{\mu }}= & {} -f_2^4\bigg (\frac{199}{15} +\frac{N_V}{5}+\frac{N_f}{20}+\frac{N_s}{60} \bigg )\nonumber \\&(\text { for } f_0\rightarrow \infty \text { and } \xi _{ab}\rightarrow -\frac{1}{6} \delta _{ab}). \end{aligned}$$

This reproduces the result obtained in [41–44] with a dedicated computation in the gauge of Eq. (13), where only the traceless part of the graviton propagates; see Eq. (16). Then the one-loop RGE for all other parameters can be obtained from the agravity RGE, listed in the appendix, by dropping those for $f_0$ and $\xi _{ab}$, as well as the terms involving $f_0$ and $\xi _{ab}+\delta _{ab}/6$ from the remaining RGE. The result is

25 $$\begin{aligned} (4\pi )^2 \frac{\mathrm{{d}}Y^a}{\mathrm{{d}}\ln \bar{\mu }}= & {} \frac{1}{2}(Y^{\dagger b}Y^b Y^a + Y^a Y^{\dagger b}Y^b)+ 2 Y^b Y^{\dagger a} Y^b \nonumber \\&+ Y^b \,\mathrm{Tr}(Y^{\dagger b} Y^a) - 3 \{ C_{2F} , Y^a\} + \frac{15}{8}f_2^2 Y^a, \end{aligned}$$

26 $$\begin{aligned} (4\pi )^2 \frac{\mathrm{{d}}\lambda _{abcd}}{\mathrm{{d}}\ln \bar{\mu }}= & {} \sum _{\mathrm{perms}} \bigg [\frac{1}{8} \lambda _{abef}\lambda _{efcd}\nonumber \\&+ \frac{3}{8} \{\theta ^A,\theta ^B\}_{ab}\{\theta ^A ,\theta ^B\}_{cd} -\,\mathrm{Tr}\, Y^a Y^{\dagger b} Y^c Y^{\dagger d} \nonumber \\&+\frac{5}{288} f_2^4 \delta _{ab}\delta _{cd}+ \lambda _{abcd}\nonumber \\&\times \bigg [ \sum _{k=a,b,c,d} (Y_2^k-3 C_{2S}^k)+ 5 f_2^2\bigg ], \end{aligned}$$

for $f_0\rightarrow \infty$ and $\xi _{ab}\rightarrow -\frac{1}{6} \delta _{ab}$, where $Y_2^k$, $C_{2S}^k$ and $C_{2F}$ are defined in Eq. (51). We do not know of any previous determinations of the RGE in (25) and (26). We do not show the RGE of the gauge couplings because they are not modified by the gravitational couplings (see the first paper in [41–44] and [4, 45, 46]).

### Anomalous generation of $1/f_0^2$

However, the fact that $f_2$ and other gauge, Yukawa and quartic couplings start having non-vanishing $\beta$-functions means that the conformal-gravity computation becomes inconsistent when going to higher orders. The conformal mode of the agraviton, $\sigma$, is a decoupled degree of freedom in the classical Lagrangian of conformal gravity. At quantum loop level, $\sigma$ starts coupling to all terms that break scale invariance proportionally to their $\beta$-functions, so that $\sigma$ can no longer be gauged away.

Once $\sigma$ couples to other particles, they can propagate in loops within Feynman diagrams containing, as external states, $\sigma$ only. This describes how the $R^2$ term is generated at a loop level sufficiently high for the diagram to contain running couplings. The result can be written in terms of $\beta$-functions through the aid of consistency conditions obtained by formally promoting the couplings to fields, including the gravitational coupling. A scalar quartic $\lambda$ starts contributing at $\lambda ^5$ order [47, 48]; a gauge interaction starts contributing at $g^6$ order [49, 50]; the effect of scalar quartics, Yukawa and gauge couplings was computed in [51] in parity-invariant theories. The final result can be written as an RGE for $1/f_0^2$:

27 $$\begin{aligned} \frac{\mathrm{{d}}}{\mathrm{{d}}\ln \bar{\mu }}\frac{1}{f_0^2}= & {} \frac{b_1 b_2 N_V}{18} \frac{g^6}{(4\pi )^8} + \frac{1}{25920(4\pi )^{12}} \nonumber \\&\times \left( 6 \lambda _{abcd}\lambda _{cdmn}\lambda _{mnpq}\lambda _{aprs}\lambda _{bqrs} \right. \nonumber \\&+12 \lambda _{abcd}\lambda _{cdmn}\lambda _{mrpq}\lambda _{bspq}\lambda _{anrs} \nonumber \\&\left. -\lambda _{acdm}\lambda _{bcdm}\lambda _{anrs}\lambda _{bnpq}\lambda _{rspq}\right) + \cdots \end{aligned}$$

in the limit $f_0\rightarrow \infty$ and $\xi _{ab}\rightarrow - \delta _{ab}/6$. We have written explicitly the leading gauge contribution assuming, for simplicity, a gauge group *G* with a single gauge coupling *g*, $N_V$ vectors and $N_f$ Weyl fermions in the same representation *R* of *G*: $b_1$ and $b_2$ are the usual one-loop and two-loop $\beta$-function coefficients for *g*, precisely defined as $\mathrm{{d}}g/\mathrm{{d}}\ln \bar{\mu } = -b_1g^3/(4\pi )^2 - b_2 g^5/(4\pi )^4 + \cdots$ and given by [52]^9^ We also have

30 $$\begin{aligned} b_1= & {} \frac{11}{3} C_{2G} -\frac{2}{3} T_FN_f, \nonumber \\ b_2= & {} \frac{34}{3} C_{2G}^2 -\frac{10}{3}C_{2G}T_F N_f-2 C_{2F}T_FN_f . \end{aligned}$$

The gauge contribution to $1/f_0^2$ can be either positive or negative, depending on the field content. For example, in the SM one has $N_V=3$, $b_1=19/6$ and $b_2=-\,35/6$ for $\,\mathrm{SU}(2)_L$ and $N_V=8$, $b_1=7$ and $b_2 =26$ for $\,\mathrm{SU}(3)_c$. The quartic of the Higgs doubled *H*, defined by the potential $\lambda _H | H|^4$, contributes to the RGE for $1/f_0^2$ as $416 \lambda ^5_H/5(4\pi )^{12}$, which is sub-dominant with respect to the gauge contributions. Integrating the gauge contribution alone from infinite energy down to a scale where $g\ll 1$, one finds $1/f_0^2 \simeq - b_2 N_V g^4/72(4\pi )^6$.

The $\, \cdots \,$ in Eq. (27) denote extra terms due to Yukawa couplings (partially computed in [51]) and to gravitational terms (never computed and presumably first arising at order $f_2^6$). The full unknown expression might perhaps take the form of a $\beta$-function of some combination of couplings, given that the Weyl symmetry is not broken when all $\beta$-functions vanish. Barring this exception, which seems not to be relevant (nature is neither described by a free theory nor by a conformal theory), Eq. (27) means that conformal gravity is not a complete theory: at some loop level, quantum corrections start generating back the extra couplings $f_0$ and $\xi _{ab}$ present in agravity.

One important aspect of Eq. (27) is that its right-hand side vanishes when all couplings sit at a fixed point, where all $\beta$-functions vanish. This tells us that the $f_0\rightarrow \infty$ limit is consistent when the other couplings on the right-hand-side approach a fixed point.

### Anomalous generation of $\xi +1/6$

Non-conformal $\xi$-couplings are generated at one-loop by the gravitational coupling $f_2$. Starting from $\xi = -1/6$ at infinite energy, $f_2$ induces a negative value of

31 $$\begin{aligned} f_0^2(\xi +1/6) \sim - {{{\mathcal {O}}}}(f_2^2) \end{aligned}$$

at finite energy. However, as argued later, naturalness demands $f_2\lesssim 10^{-8}$. At perturbative level, $f_0$ alone does not generate $\xi +1/6$. Extra anomalous contributions to $\xi +1/6$ are first generated at order $y^6/(4\pi )^6$, $y^2\lambda ^2/(4\pi )^6$, $\lambda ^4/(4\pi )^8$ in the Yukawa couplings *y* and in the scalar quartics $\lambda$ (see eqs. (6.33) and (7.22) of [51], where individual terms have different signs; see also [47, 48]). For example the quartic couplings alone contribute as

32 $$\begin{aligned} \frac{\mathrm{{d}}\zeta _{ab}}{\mathrm{{d}}\ln \bar{\mu }}= & {} \frac{1}{18(4\pi )^8} \nonumber \\&\times \, \bigg ( \frac{1}{6} \lambda _{cpqr} \lambda _{dpqr} \lambda _{cmna}\lambda _{dmnb} + \lambda _{pqmn}\lambda _{pqcd} \lambda _{cmra} \lambda _{dnrb} \nonumber \\&\quad - \lambda _{rpqd}\lambda _{rmnc} \lambda _{dmna} \lambda _{cpqb} \bigg ) + \cdots \end{aligned}$$

for $f_0\rightarrow \infty$ and $\xi _{ab}\rightarrow - \delta _{ab}/6$, where $\cdots$ denote the contribution of the other couplings. In the SM Higgs case this contribution is $\mathrm{{d}}\xi _H/\mathrm{{d}}\ln \bar{\mu } =48 \lambda ^4_H/(4\pi )^8+\cdots$, having written the potential as $\lambda _H |H|^4$ and the non-minimal coupling to gravity as $-\xi _H |H|^2 R$.

It is important to note that the right-hand-side of Eq. (32) vanishes when all couplings sit at a fixed point, where all $\beta$-functions vanish. This tells us that the $f_0\rightarrow \infty$ limit is consistent when at the same time $\zeta _{ab}\rightarrow 0$ and the other couplings approach a fixed point. In this precise limit the conformal mode decouples from the rest of the degrees of freedom.

## The conformal mode of the agraviton

So far we have shown that a large self-coupling $f_0$ of the conformal mode of the agraviton does not affect the rest of physics, provided that the non-minimal couplings $\xi$ of scalars go to the conformal value and the remaining couplings approach a fixed point. We next address the big issue: what happens to the conformal mode of the agraviton when $f_0$ is big?

The one-loop agravity RGE for $f_0$, Eq. (3), is valid for $f_0\ll 1$ and shows that a small $f_0$ grows with energy. In general, when a dimensionless coupling behaves in this way, three qualitatively different things can happen depending on the non-perturbative behavior of the $\beta$-function,

33 $$\begin{aligned} \frac{\mathrm{{d}}f_0}{\mathrm{{d}}\ln \bar{\mu }} = \beta (f_0). \end{aligned}$$

1. If $\beta (f_0)$ grows at large $f_0$ faster than $f_0$, then $\int ^\infty \mathrm{{d}}f_0/\beta (f_0)$ is finite and $f_0$ hits a Landau pole at finite energy. The theory is inconsistent.^10^
2. If $\beta (f_0)$ vanishes for some $f_0=f_0^*$, then $f_0$ grows to $f_0^*$, entering into asymptotic safety.
3. If $\beta (f_0)$ remains positive but grows less than or as $f_0$, then $f_0$ grows to $f_0=\infty$ at infinite energy.^11^

In order to study what happens at large $f_0$, we can ignore all other couplings and focus on the conformal mode of the agraviton. We can choose a conformally flat background $g_{\mu \nu }(x) = e^{2\sigma (x)} \eta _{\mu \nu }$, as the background does not affect the UV properties of the theory. Recalling Eq. (8), the action for the conformal mode only is

34 $$\begin{aligned} S= & {} \int \mathrm{{d}}^4x \sqrt{|\det g|} \frac{R^2}{6f_0^2} = \frac{6}{f_0^2} \int \mathrm{{d}}^4x (e^{-\sigma } \Box e^{\sigma })^2 \nonumber \\= & {} \frac{6}{f_0^2} \int \mathrm{{d}}^4x [\Box \sigma + (\partial \sigma )^2]^2 . \end{aligned}$$

The field $\sigma$ has mass dimension 0, and its action in Eq. (34) respects the following symmetries: shifts $\sigma (x)\rightarrow \sigma (x) + \delta \sigma$; Poincaré invariance; scale invariance; invariance under special conformal transformations:

35 $$\begin{aligned} \sigma (x)\rightarrow & {} \sigma (x') - 2 c\cdot x,\nonumber \\ x'_\mu= & {} x_\mu + c_\mu x^2 - 2 x_\mu (c\cdot x), \end{aligned}$$

at first order in the infinitesimal constant vector $c_\mu$. Conformal invariance here appears as a residual of the reparametrization invariance of the gravitational theory: it is present because conformal transformations are those reparametrizations that leave the metric invariant, up to an overall scale factor. Being a residual of reparametrization invariance, this symmetry is non-anomalous, up to the usual scale anomaly. No other action is compatible with these symmetries. Taking into account that $\mathrm{{d}}^4x = (1+8c\cdot x) \mathrm{{d}}^4 x'$, the single terms in the action of Eq. (34) vary under a conformal transformation as

36a $$\begin{aligned}&\delta \int \mathrm{{d}}^4x \, (\partial \sigma )^4 = 8\int \mathrm{{d}}^4x [ - c\cdot \partial \sigma (\partial \sigma )^2], \end{aligned}$$

36b $$\begin{aligned}&\delta \int \mathrm{{d}}^4x \, (\partial \sigma )^2\Box \sigma =4 \int d^4x [ c\cdot \partial \sigma (\partial \sigma )^2 - c\cdot \partial \sigma \Box \sigma ], \end{aligned}$$

36c $$\begin{aligned}&\delta \int \mathrm{{d}}^4x\, (\Box \sigma )^2 =8 \int \mathrm{{d}}^4x [ c\cdot \partial \sigma \Box \sigma ] , \end{aligned}$$

such that the combination in Eq. (34) is invariant.^12^ We verified, at tree level, that the scattering amplitudes vanish, in agreement with the Coleman–Mandula theorem.

For small $f_0$ one can compute the theory perturbatively around the four-derivative kinetic term $(\Box \sigma )^2$. As discussed in Sect. 2, this can be equivalently formulated as an SO(2)-invariant scalar $\Omega$ with a quartic coupling. This shows that UV-divergent quantum corrections preserve the form of the action, such that the quantum action is given by

37 $$\begin{aligned} \Gamma = Z(f_0) \, S +\hbox {finite effects}. \end{aligned}$$

Indeed, in the scalar theory with the field $\Omega$ and the simple quartic coupling all divergences can be reabsorbed by renormalizing $f_0^2$ (which in that formulation represents the quartic coupling) and the field, $\Omega$. Going back to the formulation in terms of $\sigma$, both renormalizations (of $f_0$ and of $\Omega$) can be expressed in terms of a common rescaling of the action, which is what appears in Eq. (37).

The common UV-divergent factor $Z(f_0)$ renormalizes equally all terms in the action, such that it can be seen as an RGE running of $f_0$, which we give here up to two loops:

38 $$\begin{aligned} \frac{\mathrm{{d}} f_0^2}{\mathrm{{d}}\ln \bar{\mu }} = \frac{1}{(4\pi )^2} \frac{5}{6} f_0^4 - \frac{1}{(4\pi )^4} \frac{5}{12} f_0^6 +\cdots . \end{aligned}$$

The one-loop term reproduces the corresponding term in the full gravitational computation, Eq. (3), while the two-loop term was never obtained before. The Weyl anomaly, mentioned in Sect. 2, affects this RGE only at higher loop level. The reason is that the $\beta$-functions are already one-loop effects, so that one needs at least two vertices and one extra loop to get a contribution from the anomaly. This remark not only applies to pure anomalous effects, but also to mixed $f_0$-anomaly contributions; in the latter case, indeed, a couple of internal $\sigma$-lines should be converted to the particles which $\sigma$ couples to through the anomaly and again at least two vertices proportional to $\beta$-functions and one extra loop are needed.

When $f_0$ grows the path integral receives contributions from fluctuations of $\sigma$ with larger and larger amplitude, probing the terms in the action of Eq. (34) with higher powers in $\sigma$. For large $f_0$ the action becomes dominated by the $(\partial \sigma )^4$ term that has the highest power of $\sigma$, while the kinetic term becomes negligible. This can happen because all terms in the action have the same number of derivatives. For example, a field configuration $\sigma (r) = \sigma _0 e^{-r^2/a^2}$ contributes as $S \sim (\sigma _0 + \sigma _0^2)^2/f_0^2$, independently of the scale *a*, such that for $f_0\gtrsim 1$ the path integral is dominated by the second term.

In the limit $f_0\rightarrow \infty$ the action *S* simplifies to

39 $$\begin{aligned} S_\infty = \frac{6}{f_0^2}\int \mathrm{{d}}^4x\, (\partial \sigma )^4. \end{aligned}$$

Although for large $f_0$ the theory is non-perturbative in $f_0$, one can still develop an analytical argument to show the absence of a Landau pole of $f_0$, as we now discuss. The action in Eq. (39) acquires new symmetries: $S_\infty$ is $\mathbb {Z}_2$-invariant ($\mathbb {Z}_4$-invariant if complexified); furthermore, being the term of *S* with the highest power of $\sigma$, it is invariant under the homogeneous part of the transformation in Eq. (35), while the other two terms, $(\partial \sigma )^2\Box \sigma$ and $(\Box \sigma )^2$ or any combination of them, are not. Symmetries imply that the quantum action $\Gamma _\infty$, which includes the classical and UV-divergent quantum corrections, is fully described by $\Gamma _\infty = Z_\infty S_\infty$, where $Z_\infty$ is a constant, related to the $Z(f_0)$ in the full theory as $Z_\infty =\lim _{f_0\rightarrow \infty }Z(f_0)$. This constant must equal unity, $Z_\infty =1$ because the theory is classical at large field values, for which $S_\infty \gg 1$, and because its form at all field values is fixed by symmetries. The theory with action $S_\infty$, despite being interacting, behaves as a free theory, in the sense that the quantum action does not receive divergent corrections.

This shows that, in the full theory, $f_0$ can flow to large values without hitting Landau poles: $\beta (f_0) ={{\mathcal {O}}}(1/f_0)$ at $f_0 \gg 1$. Having distilled the non-perturbative dynamics of the conformal mode of the agraviton in a simple action, Eq. (34), it seems now feasible to fully clarify its dynamics. We have shown that it hits no Landau poles, excluding case 1. of the initial list. The theory at $f_0 \gg 1$ should be computable by developing a perturbation theory in $1/f_0$. We have not been able of excluding case 2: a vanishing $\beta (f_0)$ at $f_0 \sim 4\pi$. Non-perturbative numerical techniques seem needed to determine the behavior of the theory at the intermediate energy at which $f_0 \sim 4\pi$, although this currently needs adding a regulator that breaks the symmetries of the theory (such as a lattice or a momentum averager [56–58]), obscuring possible general properties (such as the sign of $\beta (f_0)$) that could follow from the positivity of the symmetric action in Eq. (34).

The letter ‘*a*’ in the name ‘conformal mode of the *a*graviton’ reminds us that our field $\sigma$ contains two degrees of freedom because its action contains four derivatives, while the usual ‘conformal mode of the graviton’ obtained from the Einstein action only contains one degree of freedom. More precisely, the Einstein term alone, $-\frac{1}{2} {\bar{M}}_{\mathrm{Pl}}^2R$, where $\bar{M}_\mathrm{Pl}$ is the reduced Planck mass, gives a negative kinetic term $3{\bar{M}}_\mathrm{Pl}^2 \Omega _L \Box \Omega _L$ for $\Omega _L =e^\sigma$; see Eq. (7). Summing the Einstein term with $R^2/6 f_0^2$, the four-derivative conformal mode of the agraviton $\sigma$ splits into a physical mode with positive kinetic term and mass $M_0 = f_0 \bar{M}_{\mathrm{Pl}}/\sqrt{2}$ for $f_0\ll 1$, and the usual massless Einstein term, which is reparametrization dependent.^13^ To see this, it is convenient to use the form of the action where $\sigma$ is rewritten in terms of two fields with two derivatives, $\Omega _L$ and $\Omega _R$ (see Sect. 2). Adding to the previous discussion the Planck mass the Lagrangian becomes

40 $$\begin{aligned} \mathscr {L}= -2\Omega _R \Box \Omega _L - \frac{1}{6} f_0^2 \Omega _L^2 \Omega _R^2 + 3 \bar{M}_\mathrm{Pl}^2 \Omega _L \Box \Omega _L. \end{aligned}$$

**Fig. 1:**
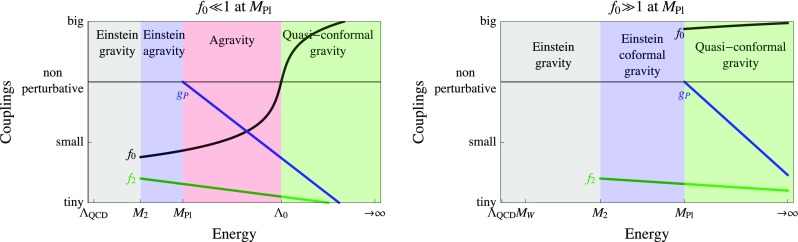
RGE running of the main dimensionless couplings $f_0, f_2, g_P$ in the two possible scenarios that do not lead to unnaturally large corrections to the Higgs mass: $f_0 , f_2 \ll 1$ at the Planck scale (left), $f_2 \ll 1$ and $f_0 \gg 1$ at the Planck scale (right). Here $M_{\mathrm{Pl}}$ is the Planck mass, $M_2\equiv f_2 \bar{M}_\mathrm{Pl}/\sqrt{2}$ is the graviton ghost mass, $\Lambda _{0}$ is the RGE scales at which $f_{0}\sim 4\pi$.

We expand in fluctuations around the minimum, $\Omega _R =0$ and $\Omega _L=1$, where we arbitrarily choose unity in order to keep the metric as $\eta _{\mu \nu }$, while other values would correspond to a different unit of mass. Then the quadratic part of the action can be diagonalized by defining $\Omega _L = 1 + (\alpha +\beta )/\sqrt{3}\bar{M}_\mathrm{Pl}$, $\Omega _R = \sqrt{3} M\beta$, where $\alpha$ is the Einstein ghost and $\beta$ is the massive scalar component of the graviton. The result is

41 $$\begin{aligned}&\mathscr {L}= \alpha \Box \alpha + \beta (-\Box - M_0^2)\beta -V\qquad \hbox {with}\nonumber \\&V = \frac{1}{6} f_0^2 \beta ^2 (\alpha +\beta ) (\alpha +\beta +2\sqrt{3}\bar{M}_\mathrm{Pl}). \end{aligned}$$

## Scenarios compatible with naturalness of the Higgs mass

In the following we discuss implications of case 3. Qualitatively different scenarios can arise, depending on the ordering between the key scales:

- $\Lambda _0$, the energy scale at which the self-coupling of the conformal mode equals $f_0 \sim 4\pi$, with $f_0 \ll 4\pi$ at $E \ll \Lambda _0$ and $f_0 \gg 4\pi$ at $E\gg \Lambda _0$.
- $\Lambda _2$, the energy scale at which the graviton self-coupling equals $f_2 \sim 4\pi$, with $f_2 \ll 4\pi$ at $E \gg \Lambda _2$.
- The Planck scale. As this is the largest known mass scale, in the context of dimensionless theories it can be interpreted as the largest dynamically generated vacuum expectation value or condensate.

The scales $\Lambda _{0,2}$ can be physically realized in nature (like the scale $\Lambda _{\mathrm{QCD}}$ at which $\,\mathrm{SU}(3)_c$ becomes strong) if they are larger than the Planck scale. Otherwise they are not realized (like the scale at which $\,\mathrm{SU}(2)_L$ would have become strong, if symmetry breaking had not occurred at a higher energy) and we use $\Lambda _{2}\ll M_{\mathrm{Pl}}$ to denote $f_{2} \ll 1$ at $M_{\mathrm{Pl}}$ where $M_{\mathrm{Pl}}$ is the Planck mass.

In this section we adopt Higgs mass naturalness as a criterion to limit the possible speculations. For example, the simplest possibility in which the Planck scale is identified with $\Lambda _2$ or $\Lambda _0$ leads to unnaturally large physical corrections to the Higgs mass from gravity. Naturalness demands $f_2 \ll 1$ at the Planck scale, while $f_0$ can be either very small or very large, giving rise to two natural possibilities shown in Fig. 1: $f_0\ll 1$ at $M_{\mathrm{Pl}}$ (left panel) and $f_0 \gg 1$ at $M_{\mathrm{Pl}}$ (right).

### $f_0 \ll 1$ at the Planck scale

The first possibility is the one considered in [4], which showed that the Planck mass can be dynamically generated, within a dimensionless theory, from a dynamically induced vacuum expectation value of a fundamental scalar $S = (s+i s')/\sqrt{2}$. The part of the dimensionless Lagrangian involving *S* and the SM Higgs doublet *H* is

42 $$\begin{aligned} \mathscr {L}= & {} \bigg [ |D_\mu S|^2 - \lambda _S |S|^4 -\xi _S |S|^2 R\bigg ]\nonumber \\&+ \bigg [ |D_\mu H|^2 -\lambda _H |H|^4 - \xi _H |H|^2 R \bigg ]\nonumber \\&+ \,\lambda _{HS}|S|^2 |H|^2. \end{aligned}$$

Provided that $\lambda _S$ runs in such a way that it vanishes at the same scale at which its $\beta$-function vanishes, *s* gets a vacuum expectation value with cosmological constant tuned to zero, and ${\bar{M}}_{\mathrm{Pl}}^2 = \xi _S \langle s\rangle ^2$ is positive provided that the parameter $\xi _S$, renormalized at the Planck scale, is positive. An unpleasant feature of the model is that the mixed quartic $\lambda _{HS}$ must be very small, in order to avoid inducing an unnaturally large contribution to the Higgs mass ($M_h^2 = \lambda _{HS} \langle s\rangle ^2$, which appears in the potential as $- M_h^2 |H|^2/2$). References [4, 6, 7] showed that $\lambda _{HS}$ can be naturally small, despite being generated at loop level through gravity loops as

43 $$\begin{aligned} (4\pi )^2 \frac{\mathrm{{d}}{\lambda _{HS}}}{\mathrm{{d}}\ln \bar{\mu }}= & {} -\,\xi _H\xi _S [ 5f_2^4 + 36 {{\tilde{\lambda }}}_H {{\tilde{\lambda }}}_S] \nonumber \\&+ \cdots \qquad (f_0 \ll 1), \end{aligned}$$

where ${{\tilde{\lambda }}}_{S} \equiv f_0^2 (\xi _S+1/6)$ and ${{\tilde{\lambda }}}_H \equiv f_0^2(\xi _H+1/6)$ are the couplings that appear in the perturbatively equivalent formulation of agravity of Eq. (10), where $f_0$ and $\xi _{H,S}$ become quartic couplings with an extra scalar $\vec \Omega$. The Higgs mass is natural if $f_{0,2} \lesssim 10^{-8}$. The above scenario needs to be reconsidered:

- Is naturalness still satisfied, or $f_0$ becoming strongly coupled at the energy scale $\Lambda _0$ generates a ${{\tilde{\lambda }}}_{H,S}$ of the same order?
- Can one get $\xi _S > 0$ at the Planck scale starting from $\xi _S = -1/6$ at infinite energy?

A peculiar RG running behavior at a very large scale, such as $\Lambda _0\gtrsim 10^{10^{16}}\,\mathrm{GeV}$, does not imply perturbative contributions to scalar masses of the same order, as long as no new physics nor vacuum expectation values nor condensates develop at that scale [25]. Non-perturbative ultra-Planckian contributions to the cosmological constant and the Planck mass from a $f_0\sim 4\pi$ are forbidden by the global shift symmetry $\sigma \rightarrow \sigma + \delta \sigma$. Planckian corrections to the cosmological constant remain unnaturally large as usual.

The answer to (a) seems to be positive: as shown in Sect. 2 perturbative corrections in $f_0$ behave like quartic scalar couplings, and thereby renormalize the ${{\tilde{\lambda }}}_{H,S}$ couplings (mixed quartics between the scalars and the conformal mode of the graviton) only multiplicatively, like in the one-loop RGE, Eq. (50d). The same happens at $f_0 \gg 1$: non-vanishing ${{\tilde{\lambda }}}_{H,S}$ are only generated by $f_2$ (see Eq. (31)) and by the multi-loop anomalous effects discussed in Sect. 3. Non-perturbative corrections in $f_0 \sim 4\pi$ presumably too renormalize ${{\tilde{\lambda }}}_{H,S}$ only multiplicatively, as the scalars *H*, *S* are not involved in the strong self-coupling of the conformal mode of the graviton.

Concerning issue (b), the answer can be positive in a theory where $\xi _S$ is very close to $-1/6$ around and above the energy scale $\Lambda _0$, and a positive $\xi _S$ is only generated through anomalous running (see e.g. Eq. (32)) at a much lower energy where $f_0 \ll 1$ by some matter coupling becoming non-perturbative.

Given that non-perturbative physics seems anyhow necessary, we propose here a simpler mechanism for the generation of the Planck mass that relies on a new strong coupling $g_P$, rather than on a perturbative coupling $\lambda _S$. Without introducing any extra scalar *S* (and thereby bypassing the issue of a small $\lambda _{HS}$), the Planck scale can be induced by a new gauge group *G* (under which the Higgs is neutral) with a gauge coupling $g_P$ that runs to non-perturbative values around the Planck scale, such that condensates *f* are generated. This is shown as blue curve in Fig. 1. This scenario can be very predictive, as one coupling $g_P$ dominates the dynamics. The sign of $M_\mathrm{Pl}^2$ is predicted; however, it is not determined by dispersion relations and seems to depend on the detailed strong dynamics of the model (gauge group, extra matter representations) [63–68].

One has the desired $M_{\mathrm{Pl}}^2>0$ provided that the theory admits an effective-theory approximation where the effect of the strong dynamics is dominantly encoded in a mixing of the graviton with a composite spin-2 resonance, analogously to how a photon/$\rho$ mixing approximates QCD effects. Then the relevant effective Lagrangian for the graviton $h_{\mu \nu }$ and the spin-2 resonance is

44 $$\begin{aligned} \mathscr {L}_{\mathrm{eff}}= & {} - \frac{M^2}{2} R_\rho +f^4[ a (h_{\mu \nu }-\rho _{\mu \nu })^2 \nonumber \\&+ (h_{\mu }^{\,\, \,\mu } - \rho _{\mu }^{\,\, \,\mu })^2] +{{\mathcal {O}}}(\partial ^4 h_{\mu \nu })+\mathcal{O}(\partial ^4 \rho _{\mu \nu }). \end{aligned}$$

The first term is the positive quadratic kinetic energy for the spin-2 resonance generated by strong dynamics; we wrote it as a ‘curvature’ $R_\rho$ multiplied by some positive $M^2>0$. The second term is a mass term, which presumably approximatively has Fierz–Pauli form, $a\approx 1$.^14^ Next, we integrate out $\rho _{\mu \nu }$ obtaining an effective action for the graviton $h_{\mu \nu }$. At leading order in derivatives one simply has $\rho _{\mu \nu }=h_{\mu \nu }$, irrespectively of the precise form of the mass term. Thereby the resulting effective action is the Einstein action, with ${\bar{M}}_{\mathrm{Pl}}^2=M^2$.

Furthermore, the strong dynamics generates at the same time a cosmological constant. In a theory with no matter charged under *G* it is negative and of order $M_{\mathrm{Pl}}^4$:

45 $$\begin{aligned} V=\frac{T_{\mu }^{\mu }}{4}= \frac{\partial _\mu {\mathscr {D}}^\mu }{4}=\frac{1}{4} \frac{\beta _{g_P}}{2g_P} \langle F_{\alpha \beta }^{A2}\rangle \end{aligned}$$

where ${\mathscr {D}}_\mu$ is the anomalous dilatation current and $\beta _{g_P} <0$.

This large contribution to the cosmological constant can be avoided if the theory also includes a Weyl fermion $\lambda$ in the adjoint of the gauge group *G*, such that the most general dimensionless action,46is accidentally supersymmetric in its strongly coupled sector. With this particle content $\langle F_{\alpha \beta }^{A2} \rangle =0$ vanishes, being the *D*-term of an accidental unbroken global supersymmetry, while the fermion condensate can be computed [70–72].

The Higgs has no renormalizable interaction with the strong sector at the Planck scale: it is only generated through gravitational loops, between the Planck mass and the masses $M_{0,2}$ of the extra components of the agraviton. The one-loop RGE for the Higgs mass in this regime was computed in [4], and the contribution proportional to $\bar{M}_\mathrm{Pl}^2$ is

47 $$\begin{aligned} (4\pi )^2\frac{\mathrm{{d}} }{\mathrm{{d}}\ln \bar{\mu }} {M_h^2}= & {} -\xi _H [5f_2^4+f_0^4(1+6\xi _H)]\nonumber \\&{\bar{M}}_{\mathrm{Pl}}^2 + \cdots \qquad \hbox {for}\qquad M_{0,2}< {\bar{\mu }} < M_{\mathrm{Pl}}\nonumber \\ \end{aligned}$$

where $\cdots$ are contributions that are not dangerous from the point of view of naturalness. In Appendix B we write the one-loop RGE for the most general massive parameters.

### $f_0\gg 1$ at the Planck scale

A simpler alternative that avoids having a very large RGE scale at which $f_0$ crosses $4\pi$ is that $f_0$ is still large at the Planck scale and never gets small.

The conformal mode of the agraviton only has small anomalous couplings, until its dynamics suddenly changes when some vacuum expectation value or condensate is first generated. We assume that the largest such effect is the Planck mass, which can be generated in the ways discussed in the previous section. Then the tree-level Lagrangian of Eq. (41) describes how $\sigma$ splits into two-derivative modes. The SO(1,1) symmetry that prevented quantum corrections to the strongly interacting theory with $f_0\gg 1$ gets broken by $M_{\mathrm{Pl}}$.

The physical difference with respect to the previous case is that only the Einstein conformal mode of the graviton appears in the effective theory below the Planck scale down to the scale $M_2$. The RGE are those of gauge-fixed conformal gravity (see Eqs. (24), (25) and (26)). Proceeding as in Appendix B, the RGE of the Higgs mass is

48 $$\begin{aligned} (4\pi )^2\frac{\mathrm{{d}}}{\mathrm{{d}}\ln \bar{\mu }} {M_h^2}= & {} \frac{5}{6} f_2^4 {\bar{M}}_\mathrm{Pl}^2 + \cdots ,\nonumber \\&\hbox {for}\qquad M_2< {\bar{\mu }} < M_{\mathrm{Pl}}, \end{aligned}$$

which is naturally small for $f_2 \lesssim 10^{-8}$.

## Conclusions

In dimensionless gravity theories (agravity), the conformal mode of the agraviton consists of two fields: the usual conformal mode of the graviton and an extra scalar, jointly described by a four-derivative action for a single field $\sigma$, defined by $g_{\mu \nu }(x) =e^{2\sigma (x)} \eta _{\mu \nu }$. The self-interactions of the conformal mode of the agraviton are controlled by a coupling $f_0$ that is not asymptotically free. In Sect. 2 we recomputed its RGE, and we extended it at the two-loop level, by developing a formulation where $f_0^2$ becomes an extra scalar quartic coupling. In the presence of scalars, their dimensionless $\xi$-couplings to gravity become scalar quartics, and the whole agravity can be rewritten as conformal gravity plus two extra scalars with an SO(1,1) symmetry. This perturbative equivalence allowed us to recompute the one-loop RGE equations of a generic agravity theory, confirming previous results [4], writing them in an equivalent simpler form where no couplings appear at the denominator in the $\beta$-functions, extending them at two loops.

In particular, rewriting $f_0^2$ as a quartic scalar clarifies why a small $f_0$ grows with energy in any agravity theory. A Landau pole would imply that agravity is only an effective theory and that the Higgs mass receives unnaturally large corrections.

In Sects. 2, 3 and 4 we have shown that, nevertheless, agravity can be a complete theory. Agravity can be extrapolated up to infinite energy, although in an unusual way: the dimensionless coupling $f_0$ grows with energy, becomes strongly coupled above some critical RGE scale $\Lambda _0$, and can smoothly grow to $f_0\rightarrow \infty$ at infinite energy. Although we have excluded that $f_0$ has a Landau pole, i.e. that it blows up at finite energy, there is another possibility which we have not studied in the present work: $f_0$ can approach asymptotically a finite non-perturbative fixed point. Analyzing this possibility requires having control on intermediate regimes where $f_0 \sim 4 \pi$, which is beyond our current ability.

Provided that all scalars are asymptotically conformally coupled (all $\xi$-couplings must run approaching $-1/6$) and all matter couplings approach a fixed point (possibly a free one, like in QCD) in the UV, the simultaneous $f_0\rightarrow \infty$ limit turned out to be consistent. In this case and in the limit of infinite energy the conformal mode of the agraviton fluctuates freely and decouples from the rest of the theory. In the UV limit the theory can then be computed by viewing $\sigma$ as a gauge redundancy, which can be fixed with the Faddeev–Popov procedure. One then obtains conformal gravity at infinite energy. In Sect. 3 we provided the one-loop RGE at the zero order in the expansion in $1/f_0^2$ and $\xi +1/6$, including the most general matter sector.

However, the conformal symmetry is anomalous and its violation is dictated by renormalization group equations that describe how the dimensionless parameters that break conformal symmetry, $f_0$ and $\xi +1/6$, are generated at a few-loop order. As a result, at energies much above $\Lambda _0$ the conformal mode of the agraviton $\sigma$ is strongly self-coupled ($f_0\gg 1$) and fluctuates wildly, being negligibly coupled to other particles. In Sect. 4 we isolated its peculiar action and showed that, despite the strong coupling, it can be controlled through its symmetries. The action is sufficiently simple for its full quantum behavior to be simulated on a Euclidean lattice.

The anomalous multi-loop RGE which generate $1/f_0^2$ and $\xi +1/6$, are not (yet) fully known, but it is already possible to discuss the physical implications of this theory. We assume that the largest mass scale dynamically generated through vacuum expectation values or condensates is the Planck scale. Two situations discussed in Sect. 5 can lead to a scenario where the Higgs mass does not receive unnaturally large corrections. If $f_0 \ll 1$ at the Planck scale one obtains agravity at sub-Planckian energies: we wrote the most general RGE for massive parameters, and we argued that a new gauge group with a fermion in the adjoint can become strongly coupled around the Planck scale and successfully generate $\bar{M}_\mathrm{Pl}$, without generating a Planckian cosmological constant (this mechanism was never explored before in the context of agravity). Alternatively, $f_0 \gg 1$ at the Planck scale seems to be a viable possibility: in this case the scalar component of the agraviton is above the Planck scale.

## One-loop RGE in agravity

When $f_0$ and all couplings are small, the one-loop $\beta$-functions $\beta _p \equiv dp/d\ln \bar{\mu }$ of all parameters *p* of the generic agravity theory of Eqs. (4) and (5), can be conveniently written in terms of the combination of parameters that appear in Eq. (10), $\zeta _{ab} = \xi _{ab}+\delta _{ab}/6$ and

49 $$\begin{aligned} {{\tilde{\lambda }}}_{abcd}= & {} \lambda _{abcd} + 3f_0^2 (\zeta _{ab} \zeta _{cd}+ \zeta _{ac} \zeta _{bd}+\zeta _{ad} \zeta _{bc}),\nonumber \\ {{\tilde{\lambda }}}_{ab}= & {} f_0^2 \zeta _{ab},\qquad {{\tilde{\lambda }}} = f_0^2.\end{aligned}$$

The RGE are

50a $$\begin{aligned} (4\pi )^2\frac{\mathrm{{d}} f_2^2}{\mathrm{{d}}\ln \bar{\mu }}= & {} -f_2^4\bigg (\frac{133}{10} +\frac{N_V}{5}+\frac{N_f}{20}+\frac{N_s}{60}\bigg ), \end{aligned}$$

where $N_V$, $N_f$, $N_s$ are the number of vectors, Weyl fermions and real scalars (in the SM $N_V=12$, $N_f = 45$, $N_s = 4$), and

50b $$\begin{aligned} (4\pi )^2 \frac{\mathrm{{d}}f_0^2}{\mathrm{{d}}\ln \bar{\mu }}= & {} \frac{5}{3} f_2^4+5f_2^2 f_0^2+ \frac{5}{6} f_0^4 + 3 {{\tilde{\lambda }}}_{ab}{{\tilde{\lambda }}}_{ab}, \end{aligned}$$

50c $$\begin{aligned} (4\pi )^2 \frac{\mathrm{{d}}{{\tilde{\lambda }}}_{abcd}}{\mathrm{{d}}\ln \bar{\mu }}= & {} \sum _\mathrm{perms} \bigg [\frac{1}{8} {{\tilde{\lambda }}}_{abef}{{\tilde{\lambda }}}_{efcd}\nonumber \\&\quad + \frac{3}{8} \{\theta ^A,\theta ^B\}_{ab}\{\theta ^A ,\theta ^B\}_{cd} \nonumber \\&\quad -\,\mathrm{Tr}\, Y^a Y^{\dagger b} Y^c Y^{\dagger d}+\nonumber \\&+\frac{5}{288} f_2^4 \delta _{ab}\delta _{cd}+ \frac{1}{16}{{\tilde{\lambda }}}_{ab} {{\tilde{\lambda }}}_{cd}\bigg ] \nonumber \\&\quad + {{\tilde{\lambda }}}_{abcd} \bigg [5f_2^2 + \sum _{k=a,b,c,d} (Y_2^k-3 C_{2S}^k)\bigg ] , \end{aligned}$$

50d $$\begin{aligned} (4\pi )^2 \frac{\mathrm{{d}}{{\tilde{\lambda }}}_{ab}}{\mathrm{{d}}\ln \bar{\mu }}= & {} \frac{f_0^2{{\tilde{\lambda }}}_{ab}}{3} + {{\tilde{\lambda }}}_{abcd}{{\tilde{\lambda }}}_{cd} +5f_2^2{{\tilde{\lambda }}}_{ab}\nonumber \\&\quad + {{\tilde{\lambda }}}_{ab} \sum _{k=a,b} \left( Y_2^k - 3C_{2S}^k \right) +2{{\tilde{\lambda }}}_{ac}{{\tilde{\lambda }}}_{cb} \nonumber \\&\quad + \frac{5 f_2^4\delta _{ab}}{18},\nonumber \\ (4\pi )^2 \frac{dY^a}{d\ln \bar{\mu }}= & {} \frac{Y^{\dagger b}Y^b Y^a + Y^a Y^{\dagger b}Y^b}{2}\nonumber \\&\quad + 2 Y^b Y^{\dagger a} Y^b + Y^b \,\mathrm{Tr}(Y^{\dagger b} Y^a)\nonumber \\&\quad - 3 \{ C_{2F} , Y^a\} + \frac{15}{8}f_2^2 Y^a. \end{aligned}$$

The sum over “perms” runs over the 4! permutations of *abcd* and $Y_2^k$, $C_{2S}^k$ and $C_{2F}$ are defined by

51 $$\begin{aligned} \mathrm{Tr} (Y^{\dagger a}Y^b)= & {} Y_2^a \delta ^{ab}, \qquad \theta ^A_{ac} \theta ^A_{cb}= C_{2S}^{a} \delta _{ab},\nonumber \\ C_{2F}= & {} t^A t^A, \end{aligned}$$

where $\theta ^A$ and $t^A$ are the generators of the gauge group for scalars and fermions, respectively (the gauge couplings are contained in $\theta ^A$ and $t^A$).

## One-loop RGE for massive parameters in agravity

For the sake of completeness we also write the RGE for the most generic massive parameters that can be added while keeping the theory renormalizable: the reduced Planck mass $\bar{M}_\mathrm{Pl}=M_\mathrm{Pl}/8\pi$, the cosmological constant $\Lambda$, scalar squared masses $m^2_{ab}$, scalar cubics $A_{abc}$, fermion masses $M_{ij}$ defined as

52 $$\begin{aligned} \mathscr {L}_{\mathrm{massive}}= & {} - \frac{1}{2} \bar{M}_\mathrm{Pl}^2 R - \Lambda - \frac{1}{2} m^2_{ab} \phi _a \phi _b \nonumber \\&- \frac{1}{6} A_{abc}\phi _a\phi _b\phi _c -\frac{1}{2} (M_{ij}\psi _i\psi _j+\hbox {h.c.}). \end{aligned}$$

The RGE for the massive terms can be obtained from the generic dimensionless RGE by considering one neutral scalar *s* as a dummy non-dynamical variable, such that

53 $$\begin{aligned} \bar{M}_\mathrm{Pl}^2= & {} \xi _{ss} s^2 ,\qquad \Lambda =\lambda _{ssss} \frac{s^4}{4!} ,\qquad M_{ij} =Y^s_{ij} s,\nonumber \\ m^2_{ab}= & {} \lambda _{abss}\frac{s^2}{2},\qquad A_{abc}=\lambda _{abcs}s. \end{aligned}$$

This technique has been used to determine the RGE of massive parameters in generic QFT without gravity [38]. Gravitational couplings have been included in some less general models in [27, 73]. The generic RGE of massive parameters in agravity are

54a $$\begin{aligned} (4\pi )^2\frac{\mathrm{{d}} \bar{M}_\mathrm{Pl}^2}{\mathrm{{d}}\ln \bar{\mu }}= & {} \frac{1}{3} m_{aa}^2+\frac{1}{3}\,\mathrm{Tr}(M^\dagger M) +2\xi _{ab} m_{ab}^2\nonumber \\&+\left( \frac{2f_0^2}{3}-\frac{5f_2^4}{3f_0^4}+2X\right) \bar{M}_\mathrm{Pl}^2, \end{aligned}$$

54b $$\begin{aligned} (4\pi )^2\frac{\mathrm{{d}} \Lambda }{\mathrm{{d}}\ln \bar{\mu }}= & {} \frac{m_{ab}^2 m_{ab}^2}{2}- \,\mathrm{Tr}[(MM^\dagger )^2]+\frac{5f_2^4+f_0^4}{8} \bar{M}_\mathrm{Pl}^4\nonumber \\&+(5f_2^2+f_0^2) \Lambda +4\Lambda X, \end{aligned}$$

54c $$\begin{aligned} (4\pi )^2 \frac{\mathrm{{d}}M}{\mathrm{{d}}\ln \bar{\mu }}= & {} \frac{1}{2}(Y^{\dagger b}Y^b M + M Y^{\dagger b}Y^b)\nonumber \\&+ 2Y^b M^{\dagger } Y^b + Y^b \,\mathrm{Tr}(Y^{\dagger b} M) \nonumber \\&- 3 \{ C_{2F} , M\} + \frac{15}{8}f_2^2 M+MX, \end{aligned}$$

54d $$\begin{aligned} (4\pi )^2\frac{\mathrm{{d}} m^2_{ab} }{\mathrm{{d}}\ln \bar{\mu }}= & {} \lambda _{abef} m_{ef}^2+ A_{aef}A_{bef} -2 \big [\,\mathrm{Tr}(Y^{\{a} Y^{\dagger b\}} M M^{\dagger }) \nonumber \\&+\,\mathrm{Tr}( Y^{\dagger \{a} Y^{b\}} M^\dagger M) +\,\mathrm{Tr}\, (Y^a M^{\dagger }Y^b M^{\dagger })\nonumber \\&+ \,\mathrm{Tr}\, (MY^{\dagger a} MY^{\dagger b} )\big ] \nonumber \\&+\frac{5}{2} f_2^4 \xi _{ab} \bar{M}_\mathrm{Pl}^2+\frac{f_0^4}{2}\left( \xi _{ab}+6\xi _{ae}\xi _{eb}\right) \bar{M}_\mathrm{Pl}^2 \nonumber \\&+f_0^2 \left( m_{ab}^2 +3\xi _{bf} m_{af}^2+3\xi _{af}m_{bf}^2 +6 \xi _{ae}\xi _{bf} m_{ef}^2\right) \nonumber \\&+m_{ab}^2 \left[ \sum _{k=a,b} (Y_2^k-3 C_2^k)+ 5 f_2^2+2X\right] , \end{aligned}$$

54e $$\begin{aligned} (4\pi )^2\frac{\mathrm{{d}} A_{abc} }{\mathrm{{d}}\ln \bar{\mu }}= & {} \lambda _{abef}A_{efc}+ \lambda _{acef}A_{efb}+ \lambda _{bcef}A_{efa} \nonumber \\&-\,2\,\mathrm{Tr}\left( Y^{\{a}Y^{\dagger b} Y^{c\}}M^\dagger \right) - 2\,\mathrm{Tr}\left( Y^{\dagger \{c}Y^{a} Y^{\dagger b\}}M\right) \nonumber \\&+\,f_0^2\left( A_{abc}+3\xi _{af}A_{fbc}+3\xi _{bf}A_{fac}+3\xi _{cf}A_{fab}\right) \nonumber \\&+\,6f_0^2\left( \xi _{ae}\xi _{bf}A_{efc}+\xi _{ae}\xi _{cf}A_{efb}+\xi _{be}\xi _{cf}A_{efa}\right) \nonumber \\&+\, A_{abc}\left[ \sum _{k=a,b,c} (Y_2^k-3 C_2^k)+ 5 f_2^2+X\right] , \end{aligned}$$

where the curly brackets represent the sum over the permutations of the corresponding indices: e.g. $Y^{\{a} Y^{\dagger b\}}= Y^{a} Y^{\dagger b}+Y^{b} Y^{\dagger a}$. Notice that *X* is a gauge-dependent quantity, equal to

55 $$\begin{aligned} X= \frac{(3c_g^2-12c_g+13)\xi _g}{4(c_g-2)}+\frac{3(c_g-1)^2f_0^2}{4(c_g-2)^2} \end{aligned}$$

using the gauge-fixing action of [4], which depends on two free parameters $\xi _g$ and $c_g$:

56 $$\begin{aligned} S_{\mathrm{gf}}= & {} - \frac{1}{2\xi _g}\int \mathrm{{d}}^4x ~ f_\mu \partial ^2 f_\mu ,\nonumber \\ f_\mu= & {} \partial _\nu ( h_{\mu \nu } - c_g \frac{1}{2} \eta _{\mu \nu } h_{\alpha \alpha }). \end{aligned}$$

The RGE of massive parameters are gauge dependent because the unit of mass is gauge dependent. Any dimensionless ratio of dimensionful parameters is physical and the corresponding RGE is gauge-independent, as can easily be checked from Eqs. (54a)–(54e). For example [4] gave the RG equation for $M_h^2/\bar{M}_\mathrm{Pl}^2$.

## Footnotes

1 This can maybe be avoided introducing an infinite series of higher derivative terms [12], but the resulting gravity theories contain infinite free parameters and are not known to be renormalizable.

2 The ample literature of ‘ghosts’ was critically reviewed in [14]; for later work, see [15–22], where it was proposed that a four-derivative variable *q*(*t*) contains two canonical degrees of freedom (d.o.f.), $q_1 = q$ and $q_2 = \dot{q}$, with opposite time-reflection parity, such that usual *T*-even representation ($q_1 |x\rangle = x |x\rangle$ and $p_1|x\rangle =i\frac{\mathrm{{d}}}{\mathrm{{d}}x}|x\rangle$) must be combined with the *T*-odd representation ($q_2 |y\rangle = i y |y\rangle$ and $p_2|y\rangle =\frac{\mathrm{{d}}}{\mathrm{{d}}y}|y\rangle$) obtaining consistent results (positive energy, normalizable wave functions, Euclidean continuation), although the interpretation of the resulting negative norm is unclear.

3 Different statements in the literature (even recent) appear either because some previous results contained wrong signs or because some authors use computational techniques that try to give a physical meaning to power divergences, obtaining gauge-dependent and cut-off-dependent results. Claims that a run-away potential with very small $f_0$ can mimic Dark Energy do not take into account bounds on extra graviton components.

4 We omitted the topological Gauss–Bonnet term.

5 Similar remarks have been made in the context of Einstein gravity (rather than in agravity) in [33–35], where it was found that Einstein gravity is equivalent to conformal gravity plus a single conformally coupled scalar. Similar statements have been made in a different theory without the $R^2/6f_0^2$ term in [36, 37].

6 For a recent summary see [38].

7 We treated the Weyl transformation as a change of variables in field space. We could equivalently have seen it as an extra gauge redundancy. In this alternative formalism, using the Fadeev–Popov procedure to fix both diffeomorphisms and the Weyl symmetry, the gauge fixing in Eq. (15) avoids mixed terms in the ghost system; the ghosts for the Weyl gauge fixing are non-dynamical and integrating them out is equivalent to the modified diffeomorphism transformation law of the traceless graviton, Eq. (22).

8 One might hope that all couplings stay at fixed points at all energies, but this possibility is excluded because one must recover a non-conformal behavior at low energies for phenomenological reasons.

9 The group quantities $C_{2G}$, $C_{2F}$ and $T_F$ are defined as usual in terms of the generators $t^A$ in the representation *R* as follows:  For example, for the vector representation of SU(*N*) we have

10 For example, lattice simulations indicate that one scalar quartic or the gauge coupling in QED behave in this way [53–55].

11 For example, this behavior is realized if the $\beta$-function has the form $\beta (f_0) = f_0 Z(f_0)$ with $Z(f_0) = b/(f_0^2 + f_0^{-2})/(4\pi )^2$ with $b>0$. Then at low energy $f_0$ runs logarithmically towards $f_0 \rightarrow 0$, and at large energy $1/f_0$ runs logarithmically towards $1/f_0 \rightarrow 0$. Indeed, the full solution for $f_0^2>0$ is $f_0^{2} =t + \sqrt{1+t^2}$ where $t = b\ln ( \bar{\mu }/\Lambda _0)/{(4\pi )^{2}}$ and $\Lambda _0$ is the transition scale at which $f_0\sim 1$.

12 Alternatively, since conformal invariance can be seen as an inversion $x^\mu \rightarrow y^\mu = x^\mu /x^2$ followed by a translation and by another inversion, one can more simply check that the action is invariant under the inversion: $\mathrm{{d}}^4 x \rightarrow \mathrm{{d}}^4 y/y^8$, $\sigma (x)\rightarrow \sigma (y) + \ln y^2$ and $[\Box _x\sigma + (\partial _x\sigma )^2]= y^4 [\Box _y\sigma + (\partial _y\sigma )^2]$. The transformation rule of $\sigma$ under the coordinate transformation $x^\mu \rightarrow y^\mu = x^\mu /x^2$ can be obtained by recalling its general definition in (6) and that we are assuming here a conformally flat metric, i.e. $g_{\mu \nu }(x) = e^{2\sigma (x)} \eta _{\mu \nu }$.

13 Many authors refuse to view the theory with higher derivative as legitimate because of the consequent ghosts; see e.g. [59] for attempts to discard the $(\Box \sigma )^2$ term. Accepting the presence of higher derivatives allows one to describe the Weyl anomaly as ordinary RGE running of $f_{0,2}$, rather than by modifying Einstein gravity by adding a complicated ‘quantum anomalous action’ [60–62] which encodes the anomalous behavior of generic undefined theories of gravity.

14 It can be rewritten in a covariant form as the mass term resulting, in the unitary gauge, from the spontaneous symmetry breaking of general coordinate invariance acting separately on ordinary fields and on composite fields, $\hbox {GL}_h \otimes \hbox {GL}_\rho {\mathop {\rightarrow }\limits ^{f}}\hbox {GL}$ (see e.g. [69]).
